# Genetic, lifestyle and metabolic factors contributing to cardiovascular disease in the Italian population: a literature review

**DOI:** 10.3389/fnut.2024.1379785

**Published:** 2024-04-04

**Authors:** Claudia Ojeda-Granados, Elisabetta Campisi, Martina Barchitta, Antonella Agodi

**Affiliations:** Department of Medical and Surgical Sciences and Advanced Technologies “GF Ingrassia”, University of Catania, Catania, Italy

**Keywords:** chronic disease, genetic risk, susceptibility, molecular epidemiology, lifestyle, diet

## Abstract

Cardiovascular diseases (CVD) represent a major health problem worldwide. In Italy, despite the decline in CVD mortality and disability-adjusted life years recently observed, CVD remains the leading cause of death. The development of CVD has a complex and multifactorial etiology that involves environmental, lifestyle/behavioral (e.g., unhealthy diet, physical inactivity, smoking, and alcohol abuse), metabolic, and genetic factors. Although a large number of CVD susceptibility genetic variants have been identified, some seem to confer risk according to the genetic background or ethnicity of the population. Some CVD-associated polymorphisms with appreciable frequency in the Italian population may be important contributors to the development and progression of the most prevalent CVD in the population. This literature review aims to provide an overview of the epidemiology of CVD in Italy, as well as to highlight the main genetic, lifestyle/behavioral, and metabolic factors contributing to CVD risk in this population.

## 1 Introduction

Cardiovascular diseases (CVD) are the leading cause of mortality, morbidity, and disability in Europe, accounting for 45% of total deaths (1, 2). In Italy, although a decline in the CVD mortality rate has been evidenced over the last three decades, CVD remains the leading cause of mortality, morbidity, and disability (2). CVD comprises a group of heart and blood vessel disorders, including coronary heart disease (CHD) (also called ischemic heart disease −IHD or coronary artery disease −CAD), cerebrovascular disease, and peripheral artery disease, among others (3). The etiology of CVD is complex, with multiple environmental, lifestyle/behavioral (e.g., unhealthy diet, physical inactivity, smoking, and alcohol abuse), and genetic factors contributing to its development and progression (4). Lifestyle/behavioral factors often contribute to the well-known metabolic risk factors, including high systolic blood pressure, elevated total cholesterol, elevated fasting glucose, and increased body mass index (5). Genetic risk factors, on the other hand, generally comprise the presence of genetic variants or risk alleles that confer susceptibility to the development of CVD (6). A wide variety of genetic polymorphisms associated with an increased risk of CVD have been identified to date, findings that have been replicated in association studies conducted in different populations (7). However, the risk conferred by some genetic variants may be closely related to the genetic ancestry or ethnicity of the population (8). When multiple risk factors are present, they may have a synergetic or multiplicative effect on the risk of developing CVD, rather than merely an additive effect (1). Prevention of this complex disease should be based on an integrated, multidisciplinary approach to manage and control CVD risk factors and their interactions (9). Evidence suggests that simultaneous and comprehensive treatment of all cardiovascular risk factors, rather than a single factor alone, is linked to CVD burden reduction (9). Specifically, in Italy, a considerable proportion of the population smokes, is physically inactive, has a modest adherence to their traditional Mediterranean diet (MedDiet), and suffers from overweight/obesity, hypertension, dyslipidemia or type 2 diabetes mellitus (T2DM) (1, 10). Given the complexity of this disease, knowing the population-specific risk factors that contribute to their CVD risk could help to design preventive, treatment, and surveillance strategies tailored to their particular context. This review aimed to provide an overview of the epidemiology of CVD in Italy, as well as to highlight the main genetic, lifestyle/behavioral, and metabolic factors contributing to the CVD risk in this population.

## 2 Epidemiology of CVD in the Italian population

### 2.1 CVD prevalence and incidence

According to the European Cardiovascular Disease Statistics 2017, during the last two decades the absolute number of CVD cases has increased in Europe (5). In Italy, the overall crude prevalence of CVD is nearly 2 fold higher than the global prevalence (12.9% vs. 6.6%) based on epidemiological estimates derived from the Global Burden of Disease Injuries and Risk Factors Study 2017 (11). IHD and stroke are responsible for the most important CVD burden in Italy (11). More specifically, IHD and stroke show a crude prevalence of 3.6 and 1.3%, with corresponding global estimates of 1.7 and 1.4%, respectively (11). Hypertensive heart disease is also frequent in the Italian population, with a crude prevalence of 0.7% as opposed to a global prevalence of 0.2% (11). Considering instead high-risk population groups, the CAPTURE project study that integrates data on T2DM adults from 13 different European countries reports an estimated overall weighted prevalence of CVD in 2018–19 of 34.8%, while 38.8% for the Italian population (12, 13). Considering only the Italian CAPTURE cohort, atherosclerotic CVD reached a frequency of 33.1%, with CHD being the most prevalent subtype (20.8%), followed by carotid artery disease/stenosis (13.2%), cardiac arrhythmia and conduction abnormalities (7.0%), and cerebrovascular disease (5.4%) (13). In detail, the most frequent CHD types included myocardial infarction (MI), angina, coronary artery stenosis, and heart failure, while the most frequent forms of cerebrovascular disease comprised ischaemic stroke and transient ischaemic attack (13, 14).

On the other hand, according to the European Society of Cardiology (ESC) CVD 2021 statistics, middle-income ESC member countries show the highest burden of CVD, with 30% higher incidence rates compared with high-income countries (10). In fact, incidence estimates for IHD and stroke have decreased by more than 25% over the past 30 years, especially in high-income countries (10). Overall, IHD is twice as high in males than in females, while stroke has similar incidence rates in both genders (10). In Italy, the crude incidence of CVD is higher than the global incidence (0.6% vs. 0.2%) (11). In particular, IHD and stroke have crude incidence estimates of 0.07 and 0.04% in Italy, whereas the corresponding global estimates are 0.03 and 0.03% (11).

### 2.2 CVD mortality and morbidity

CVD caused 45% of all deaths in Europe and represents the leading cause of death among men in countries considered by the European Statistics on Cardiovascular Diseases 2017 (5). Similarly, according to the ESC − CVD 2021 report, CVD remains the most common cause of death, accounting for 45 and 39% of all deaths in women and men, with IHD and stroke representing the first and second most common causes of death (10). In Italy, CVD represents the leading cause of death, contributing to 34.8% of all deaths in 2017, although a decline of −53.8% in the CVD mortality rate has been recorded since 1990 (2). Compared to global estimates, CVD mortality and morbidity are lower in Italy (11). In fact, CVD accounted for 31.8% of deaths globally while 34.8% in Italy, also showing a lower age-standardized mortality rate compared to the global one (113/100,000 vs. 233.01/100,000) (11). Also in Italy, IHD and stroke represented the first and second leading cause of total CVD death contribution in 2017 (2). Specifically, IHD accounted for 15.5% of deaths and its impact on morbidity was 6.8%, whereas stroke accounted for 9.5% of deaths and its impact on morbidity was 4.3% (11). Mortality rates from IHD and stroke in Italy have been reported to be higher in men than in women (2).

## 3 Genetic determinants of CVD

### 3.1 Genetic background and susceptibility to CVD

Regarding genetic risk factors, genome-wide association studies (GWAS), genetic association studies, and high-throughput DNA sequencing technologies have led to the identification of variants in candidate genes or genomic regions associated with susceptibility for some CVD (7). For example, it has been estimated that 40–60% of the interindividual variations in CAD susceptibility are related to heritability, with specific genetic variants playing a key role in CAD pathogenesis (15, 16). The association between particular genetic variants and certain CVD has been replicated in various studies and populations. Still, some of them seem to confer risk in relation to the population’s genetic background or ethnicity (8). For instance, the association of single nucleotide polymorphisms (SNPs) at the 9p21.3 locus with CAD and MI represents one of the most replicated among diverse populations (17, 18). However, linkage disequilibrium (LD) between SNPs (e.g., rs9632884, rs10757274, rs2383206, rs1333042, rs1333040, and rs1333049) in this genomic region varies according to genetic ancestry, resulting in different SNPs being associated or not with CVD for each population (18). The rs10757274 and rs2383206 have been found to be in strong LD in Caucasians and associated with CAD in risk allele carriers (17). Interestingly, both risk alleles were also found with appreciable frequencies but not associated with CAD in a subgroup of African Americans, indicating that genetic variants may also require a specific environment to come into effect (16, 17). In individuals of European descent, the probability of carrying one or two CAD risk-associated alleles at the 9p21.3 locus is 50 and 25%, respectively (16). The association of SNPs at this locus with CAD has been confirmed even when adjusting for potential confounding covariates (e.g., age, sex, lipid levels, blood pressure, T2DM, etc.), suggesting that risk-associated alleles have an effect independent of traditional CVD risk factors (16, 17).

Besides SNPs with no apparent direct relationship to traditional risk factors, other variants with more evident relationships to gene function, influence on cardiovascular risk factors, and CVD risk have also been identified. SNPs at the 1p13.3 locus consistently associated with low-density lipoprotein cholesterol (LDL-C) levels represent an example of such a direct relationship whose association with CAD has been replicated by several studies (19, 20). The rs599839 representative of this locus has been associated with elevated LDL-C levels and CAD particularly in Caucasian European and Asian populations, but not in African Americans, in whom the A-risk allele is rare (19–22). The following section of the review describes the CVD-associated genetic variants that have been identified in a population of European ancestry, particularly in the Italian population.

### 3.2 Candidate genes and variants associated with CVD in the Italian population

CVD-associated polymorphisms with appreciable frequency in the Italian population have generally been identified in genes or regions involved in different processes such as cell adhesion, inflammation, and cellular stress processes, hemodynamic regulation, lipid traits, insulin signaling, and glucose homeostasis, thus contributing to the risk of the most prevalent CVD through different mechanisms (Table 1). The allele and genotypic frequencies of these polymorphisms in the Italian population are presented in Supplementary Table 1.

**Table 1:** Candidate genes and variants associated with cardiovascular disease risk in the Italian population.

Gene/locus	SNP names	rs number	Study population	Key findings	References
*CELSR2* (1p13.3 locus)	G > T	rs629301	Italian cohort of 2,429 patients undergoing coronary angiography	T allele associated with extension and severity of CAD, ↑ LDL-C, non-HDL-C, apoB, apoE, and apoCIII levels, ↓ HDL-C levels, independently of common risk factors	(23)
*CDKN2A-CDKN2B* (9p21.3 locus)	C > G	rs1333049	711 Italian CAD patients and 755 controls	↓ risk of CAD in G allele carriers independently of common risk factors	(8)
*APOC3* *MMP3* *SELE* 9p21.3 locus	−482C > T 1,171 5A > 6A G98T C > G	rs2854117 rs3025058 rs1805193 rs1333049	114 patients with early onset of IHD 384 patients with late onset of IHD	SNPs associated with early onset of IHD. Strong interaction for the presence of rs2854117 and rs1333049 with HTG. ↑ risk of early IHD in patients in the top tertile of multilocus GRS considering risk alleles vs. those in the bottom tertile	(24)
*LRP8*		rs7546246, rs2297660, rs3737983, R952Q rs5177	Three European-ancestry cohorts including and Italian cohort (248 MI patients +308 controls) and one Asian cohort	TACGC risk haplotype was significantly associated with familial MI in the European-ancestry cohorts including the Italian cohort. The CATAG risk haplotype was also identified among Italians	(25)
*NPR3*	−55C > A		Italian cohort of 368 early-onset ischemic stroke cases and 335 controls	Significant association of the −55 AA genotype with stroke independently of common risk factors	(26)
*TCF7L2*	T > C C > T G > T	rs7901695 rs7903146 rs12255372	Italian cohort of 154 T2DM patients and 171 healthy controls	All three SNPs associated with T2DM susceptibility, both at genotypic and at allelic level, as well as with diabetic complications (diabetic retinopathy, CVD and CAD). Carriers of CTT risk-allele haplotype showed ↑ risk of T2DM	(27)
*TP53*	Arg72Pro	rs1042522	198 Italian subjects with CAD and 129 with CVD without CAD	Significant relationship of *Pro variant with cardiac function, measured as LVEF, in subjects with CAD	(28)
*ENPP1*	K121Q	rs1044498	Prospective study (≈37 months) considering 3 Italian cohorts (330 subjects with T2DM and CAD, 141 with MI, and 266 with end-stage renal disease)	↑ hazard ratio for incidence of cardiovascular events in KQ + QQ carriers versus KK carriers considering the three cohorts combined. Polymorphism is an independent predictor of major cardiovascular events in high-risk individuals	(29)
9p21.3 locus	C > T	rs1333040	Italian cohort of 1,508 patients hospitalized for a first MI before 45y age, followed up for the occurrence of cardiovascular events	SNP significantly influenced the hazard ratio of coronary revascularization (1.38; 95% CI: 1.17–1.63 for CT and 1.90; 95% CI: 1.36–2.65 for TT carriers). It also significantly influenced the angiographic endpoint of coronary atherosclerosis progression	(30)
*PECAM1*	V125L N563S G670R 53 G > A	rs281865545 – rs1131012 –	431 Italian CAD patients and 119 controls	125 V/V, 563 N/N, and 53 G/G genotypes were associated with CAD independently of common risk factors	(31)
*TNF*	−308 G/A	rs1800629	248 Italian CAD patients and 241 controls	Significantly ↑ frequency of −308A allele in CAD patients vs. controls	(32)
*CYBA*	C242T (Tyr72His)	rs4673	276 CAD patients and 218 controls	↑ risk of early CAD and coronary stenosis progression in 242 T allele carriers	(33)
*IL6 ICAM1*	−174 G > C 469 E > K	rs1800795 rs5498	119 patients with history of ischemic stroke and 133 controls	IL-6 -174 GG and ICAM-1469 E/E genotypes were significantly associated with history of ischemic stroke. ↑ risk of stroke in subjects carrying both genotypes	(34)
*MTHFR*	677 C > T	rs1801133	655 subjects, with (433) or without (222) angiographically documented CAD	↑ risk of CAD determined by folate levels below specific thresholds combined with carrying T allele	(35)
*eNOS (NOS3)*	Glu298Asp T786 C	rs1799983 rs2070744	415 subjects undergoing coronary angiography	298Asp and 786C variants were significantly associated with occurrence and severity of CAD	(36, 37)
*APOB* *APOE* *LIPC*	XbaI E2/E3/E4 T202T (C > G)	rs693 rs429358 and rs7412	102 CAD subjects and 104 controls	ApoB Xba1 and ApoE4 variants associated with various markers of dyslipidemia and CAD Independent role observed for the LIPC T202T variant	(38)
*ACE* (17q23) *AGTR1*	I/D 287-bp A1166C	rs1799752 rs5186	205 CAD patients and 209 controls	↑ risk of CAD in the presence of ACE DD and *AGTR1* CC genotypes independently of other risk factors; stronger association with MI in ACE DD and *AGTR1* C allele carriers	(39)
*ACE*	I/D 250-bp	rs1799752	388 patients with CAD and 290 healthy subjects	*ACE* D allele was the strongest risk factor for atherosclerosis, and significantly associated with the risk of MI	(40)

CAD, coronary artery disease; LDL-C, low-density lipoprotein cholesterol; HDL-C, high-density lipoprotein cholesterol; SNP, single nucleotide polymorphisms; IHD, ischemic heart disease; GRS, genetic risk scores; MI, myocardial infarction; T2DM, type 2 diabetes; CVD, cardiovascular disease; LVEF, left ventricular ejection fraction.

#### 3.2.1 Genes involved in cell adhesion, inflammation, and cellular stress processes

Most of the polymorphisms for CVD susceptibility in the Italian population have been identified in genes that play an important role in cellular adhesion, inflammation, and cellular stress processes. The adhesion of circulating cells to the arterial surface represents an early detectable process of atherogenesis (31). Platelet endothelial cell adhesion molecule-1 (PECAM-1/CD31), involved in leukocyte transmigration and angiogenesis, is also implicated in plaque formation, thrombosis, and the development of atherosclerosis (31). In the Italian population, the *PECAM1* V125L and N563S polymorphisms, as well as the 53 G > A variant located in the 5′ untranslated region of the gene, were associated with CAD (31). In particular, the 125 V/V, 563 N/N, and 53 G/G genotypes were associated with CAD independently of conventional risk factors (31). The *eNOS* also represents a candidate gene implicated in CAD risk. Nitric oxide (NO) can inhibit several key steps in atherosclerosis, including the adhesion of platelets and leukocytes to the endothelium (36). Therefore, *eNOS* genetic variants may influence susceptibility to atherosclerosis by altering the amount of NO produced by the vascular endothelium (37). The *eNOS* Glu298Asp (rs1799983) and T786C (rs2070744) variants were significantly associated with the occurrence and severity of CAD among Italian people (36, 37). CAD risk was increased in subjects homozygous for the 786C allele independently of other common risk factors. Moreover, individuals with the Glu2983 Asp/Asp genotype and at least one 786C allele had an increased risk of CAD (36, 37).

Inflammation is also a key factor in the development of CVD. A possible role of the *TNF* − 308 G/A (rs1800629) SNP in the predisposition to the development of CAD in Italians has been evidenced (32). A significantly higher frequency of the *TNF* -308A allele was found in patients with CAD, suggesting a genetic predisposition to produce higher amounts of this proinflammatory cytokine and to develop a stronger inflammatory response contributing to CAD development (32). Additional genes involved in inflammation and cellular adhesion mechanisms include *IL6* and *ICAM1*. Polymorphisms in these genes have been associated with several atherosclerotic and ischemic disorders. In Italian patients, an increased risk of stroke was found in carriers of both the *IL6*–174GG and *ICAM1* 469E/E genotypes of the *IL6*–174 G > C (rs1800795) and *ICAM1* 469 E > K (rs5498) polymorphisms (34). Genetic variants in the *MTHFR* and *CYBA* genes have also been shown to have a role in chronic inflammatory processes and tissue stress related to CAD development (33, 35). The *MTHFR* C677T SNP (rs1801133) is widely known to impact serum homocysteine levels, a marker of inflammation, especially in inadequate folate intake (35). In Italian subjects, a gene-nutrient interaction defining an increased risk of CAD determined by folate levels below specific thresholds was evidenced among carriers of the *MTHFR* 677 T allele (35). On the other hand, CYBA (p22phox), an essential component for NADPH oxidase assembly and activation and, consequently, a relevant player in oxidative stress, was found to be associated with CAD. There is evidence that the *CYBA* C242T variant (rs4673) significantly reduces vascular NADH/NADPH oxidase activity. A significantly increased risk of early CAD and coronary stenosis progression was found in Italian 242 T allele carriers (33).

In addition, the *TP53* Arg72Pro (rs1042522) polymorphism, affecting the biochemical and functional properties of the p53-encoded protein, has shown a significant relationship with cardiac function, evidenced by a lower left ventricular ejection fraction (LVEF) in Italians carrying the *Pro allele (28). Unlike the *Arg variant with apoptosis-inducing properties, the *Pro variant results in a stronger transcriptional activator that could aggravate local coronary inflammatory lesions with a negative effect on cardiac function particularly in subjects with CAD (28).

#### 3.2.2 Genes with hemodynamic regulation function

A genetic variant (−55 C > A) in the promoter region of the *NPR3* gene, having an important role in the regulation of blood volume and pressure, has been found to influence susceptibility to cerebrovascular disease in the Italian population (26). In a cohort of Italian individuals with early-onset ischemic stroke, a significant association of the −55 AA genotype with stroke was observed independently of common risk factors (i.e., age, gender, hypertension, hypercholesterolemia, smoking habit, and T2DM) (26). Furthermore, polymorphisms in the *ACE* and *AGTR1* genes, also involved in blood pressure regulation, were found to be associated with risk for hypertension, MI, and CAD, possibly with a synergic effect (39, 40). These include the *ACE* insertion/deletion (I/D) polymorphism (rs1799752) involving a 287-bp Alu repeat sequence in intron 16 of the gene, and the A1166C (rs5186) SNP in the *AGTR1* gene (39, 40). In Italian patients, an increased risk of CAD was observed among *ACE* DD and *AGTR1* CC genotype carriers, independently of other risk factors (39). A strong association with MI was also observed in carriers of the *ACE* DD genotype and the *AGTR1* C allele, suggesting that the *ACE* D variant is associated with the development of coronary artery stenosis and the occurrence of MI and that the *AGTR1* C allele contributes synergistically to the risk of MI (39, 40).

#### 3.2.3 Genes involved in lipid traits

Polymorphisms in genes modulating serum lipid levels have been associated with an increased risk of CAD/IHD, stroke, and MI in the Italian population. In particular, the rs629301 SNP located in the intergenic region between *PSRC1* and *CELSR2* genes at 1p13.3 locus was associated with the extension and severity of CAD independently from other CVD risk factors (23). The T/T genotype correlated with higher levels of LDL-C, non-HDL cholesterol, apoB, apoE, and apoCIII, and lower HDL-C (23). Also, the *APOC3*–482 C > T (rs2854117) SNP which has shown a significant interaction with hypertriglyceridemia, has been found to be associated with early onset of IHD (24). In addition, a risk haplotype (TACGC) in the *LRP8* gene identified among cohorts of European ancestry participants, including an Italian cohort, was significantly associated with familial and early-onset CAD and MI (25). Homozygous subjects (TACGC/TACGC) showed significantly higher LDL-C levels than heterozygotes (25). Specifically, in Italians, another risk haplotype (CATAG) associated with familial MI was identified. Other genetic variants in the *APOB*, *APOE,* and *LIPC* genes have also been associated with CAD in the Italian population (38). In particular, the *APOB* XbaI (rs693) and the *APOE* ε4 variants were associated with diverse markers of dyslipidemia and CAD, while an independent role was observed for the *LIPC* T202T variant (38). Given that apolipoprotein B (ApoB) is the main component of LDL particles, *APOB* polymorphisms often contribute to CAD due to their role in regulating LDL metabolism and utilization (38). Instead, common SNPs (rs429358 and rs7412) in *APOE*, result in the main ε2, ε3 and ε4 alleles widely known for their lower, normal, and higher affinity to the LDL receptor, respectively. An increased plasma LDL-C has been associated with the Apoε4 variant and can therefore be considered potentially atherogenic. The LIPC 202G polymorphism may also be associated with higher triglyceride and lower HDL levels (38).

#### 3.2.4 Genes involved in insulin signaling and glucose homeostasis

Genetic variants in the Italian population that have been associated with CVD through the modulation of insulin signaling and glucose homeostasis have been found in the *TCF7L2* and *ENPP1* genes (27, 29). Three *TCF7L2* SNPs (rs7901695, rs7903146, and rs12255372) have shown a strong association with impaired insulin secretion, susceptibility to T2DM, and some T2DM complications (i.e., diabetic retinopathy, CVD, and CAD), both at the genotypic and allelic levels (27). Furthermore, carriers of the *TCF7L2* CTT risk-allele haplotype showed an increased risk of T2DM, compared with subjects carrying the wild-type (TCG) haplotype (27). A strong correlation was particularly found between the rs7903146 and the presence of cardiac autonomic neuropathy (27). A nonsynonymous polymorphism in the *ENPP* gene (K121Q, rs1044498), which results in inhibition of insulin receptor signaling, has been associated with insulin resistance in several but not all studies. In Italians, this variant constituted an independent predictor of major cardiovascular events in T2DM individuals carrying the KQ or QQ genotypes, an effect that was exacerbated by the presence of obesity (29).

#### 3.2.5 The 9p21.3 locus

SNPs at the 9p21.3 locus that have been studied in the Italian population include rs1333040 (C > T) and rs1333049 (C > G), the latter with variable results. In a cohort of Italian patients with early-onset MI, the presence of the rs1333040 T allele significantly influenced the progression of coronary atherosclerosis and the likelihood of coronary artery revascularization during long-term follow-up (30). On the other hand, the G allele of the rs1333049 SNP was associated with a significantly lower risk of CAD, independently of common risk factors (8). However, in a study evaluating the effect of diverse CAD and/or MI high-risk SNPs, the rs1333049 variant resulted among those strongly associated with the risk of premature IHD, suggesting that it may synergically cooperate with other cardiovascular risk factors such as hypertriglyceridemia and smoking (24). Specifically, the simultaneous presence of the risk allele and hypertriglyceridemia doubled the risk of early IHD, while the presence of smoking led to a 1.5-fold increased risk of early IHD (24).

### 3.3 Genetic risk scores for CVD

Genetic risk scores (GRS) can provide important information about the genetic component contributing to disease susceptibility, especially when considering those variants with relevant frequency per population. A study in Italian patients with early and late onset of IHD, considered a general set of 44 CAD and/or MI high-risk SNPs, identifying four genetic variants (*APOC3–*482 C > T, *MMP3* 1,171 5A > 6A, *SELE* G98T, and 9p21.3 locus rs1333049 C > G) as independent predictors of early onset of IHD (24). The combined effect of these SNPs was assessed by a GRS evidencing that the presence of high-risk alleles conferred an additive effect on cardiovascular events (24). Specifically, each risk allele was associated with a 1.3-fold higher risk of premature onset of IHD, with a gradual decrease in patients’ age as the number of risk alleles carried increased (24).

## 4 Lifestyle/behavioral factors contributing to CVD

More than 80% of the CVD burden can be attributed to potentially modifiable lifestyle/behavioral risk factors, such as diet, alcohol consumption, physical activity, and smoking (2, 41). It has been reported that only 7.3% of men and 13.0% of women in Italy have a healthy lifestyle, involving a combination of healthy dietary habits, the practice of physical activity, and the absence of smoking (1). A higher prevalence of a healthy lifestyle in both genders, with greater adherence in women than in men, is observed especially among subjects with a high educational level compared to those with a low educational level (1).

### 4.1 Diet and alcohol use

Dietary habits make the largest contribution to the risk of CVD mortality worldwide and CVD disability-adjusted life years (DALYs) across Europe (5, 10). Recent evidence suggests that dietary factors may influence mortality from CHD with one in five premature deaths being preventable with healthy dietary habits (10). In Italy, dietary habits represented the CVD risk factor with the second largest proportion of attributable CVD DALYs, preceded only by high systolic blood pressure (1). In fact, elevated systolic blood pressure and dietary habits together accounted for the largest proportion of age-standardized attributable CVD DALYs, whereas the burden of alcohol consumption declined over the last decades (1). In ESC member countries, the risk of CVD increased according to dietary habits, with a higher risk of CVD observed in high-income countries, characterized by a higher intake of sugar, trans-fatty acids, and sugar-sweetened beverages, while a lower consumption of fruits and vegetables (10). Likewise, diets rich in trans-fatty acids and red meat were associated with CHD risk and mortality, whereas substitution with polyunsaturated fats reduced CVD risk by 13% (10). High intakes of sugars and fats, especially trans and saturated fatty acids, increase the risk of atherosclerosis, while high intake of sodium increases the risk of hypertension, thus contributing to the development of CVD (10). Furthermore, high alcohol consumption, especially binge drinking, increases CVD risk by raising blood pressure and serum triglyceride levels (5). According to the WHO Global Health Observatory, the prevalence of binge drinking is considerably higher in males than females across 51 European countries (5). However, among European countries, heavy drinking was less prevalent in Italy in both genders, with a prevalence of 9% in males and 0.6% in females (5). In particular, according to the Health Examination Survey (HES) 2018–2019 within the CUORE Project of the Istituto Superiore di Sanità, more than two-thirds of the evaluated men and women had an alcohol consumption within the recommended limits according to sex and age (42).

On the contrary, dietary patterns rich in fruits, vegetables, legumes, whole grains, and lean protein sources, with low consumption of processed foods, trans-fats, sugar-sweetened beverages, sodium, and alcohol have proven cardioprotective effects (9, 10, 43). The MedDiet, rich in fruits, vegetables, legumes, nuts, whole grains, and unsaturated fatty acids, is a dietary model widely known for its positive effects on health, including CVD prevention (1, 9, 10). In the populations of the Mediterranean basin, including the Italian population, good adherence to the MedDiet is associated with a 9% reduction in CVD mortality (9, 10). In Italy, despite the strong link with gastronomic tradition, adherence to the MedDiet is rather modest (44). Considering a nationally representative sample, 31.4% of Italian adults have demonstrated low adherence, 31.3% low-to-moderate, 24% moderate-to-high, and only 13.3% high adherence to the MedDiet (45). Analysis of CVD risk markers shows that good adherence to this dietary pattern has beneficial effects on blood pressure, lipid profile, inflammation, oxidative stress, and carotid atherosclerosis (46, 47). In addition, it also influences the expression of proatherogenic genes involved in thrombosis and vascular events (46). In fact, nutritional genomic studies have demonstrated interactions between MedDiet and some genetic variants, such as the *TCF7L2* rs7903146, by reducing their adverse effect on CVD risk (46).

### 4.2 Physical activity

Regular physical activity and/or aerobic training are important preventive factors for CVD development. Evidence shows that a sedentary lifestyle raises CVD risk by increasing the risk of hypertension, high triglycerides, low HDL plasma level, T2DM, and obesity (5). One-third of adults living in ESC member countries are physically inactive, especially in high-income countries compared with middle-income ones (10). According to the Eurobarometer survey on physical activity, participation in exercise or sport is relatively low across the EU (5). On average, 42% of respondents reported never exercising or practicing sports and only 8% reported doing so at least five times a week. In Italy, 60% of respondents reported never exercising or practicing sports, and 50% reported not participating in informal physical activities either (5). In 2020, only 36.6% of Italians reported practicing any sport in their free time, of which 27.1% reported practicing it frequently, while 9.5% do it occasionally (48). On the other hand, those who only engage in some physical activity accounted for 28.1% of the population, while 35.2% reported being sedentary (48). Similarly, according to data from the Italian HES 2018–2019—CUORE Project, sedentary lifestyle during leisure time was 34 and 45% among men and women aged 35–74 years (42).

### 4.3 Smoking

Smoking is a major modifiable risk factor for CVD. Smoking may raise the risk of IHD by increasing the tendency of the blood to clot and raising blood pressure, as well as favoring atherosclerosis due to its inflammatory action on arteries. It can also decrease plasma HDL levels and exercise tolerance (5). Over the past three decades, the prevalence of smoking among men has decreased in almost all European countries (5). Across ESC member countries, CVD mortality rates decreased according to smoking prevalence reduction (10). In fact, a smaller reduction in deaths attributable to CVD has been observed in middle-income countries characterized by a high proportion of male smokers (10). In 2015, in Italy, smoking prevalence among adults aged ≥15 years was 24.8% in males and 15.1% in females (5). In 2018–2019 according to the CUORE Project HES, total cigarette current smokers were 23 and 19% of men and women aged 35–74 years (42). Furthermore, smoking was primarily responsible for the increased risk of CVD among Italians aged 40–49 years (26% smokers) in a population sample participating in the 2015 World Hypertension Day survey (49). Although smoking is dangerous at any age, the associated risk of developing CVD is closely related to the age of onset (9). Smoking cessation can reduce the risk of death due to CVD (2, 5). In particular, among heavy smokers, smoking cessation has been associated with a significantly lower CVD risk within 5 years compared with current smokers (50). However, compared with never-smokers, the risk of former smokers remained significantly elevated beyond 5 years after smoking cessation (50). Smoking cessation without subsequent weight gain is also associated with a reduced risk of CVD and mortality (51). Weight gain that may occur following smoking cessation may attenuate the reduction in CVD risk but does not attenuate the beneficial effect of smoking cessation for mortality (52).

### 4.4 Self-care management

Self-care management requires people to have adequate knowledge of health-related behavior and lifestyle, as well as awareness and perception of the risk of developing diseases. For example, optimal nutrition knowledge among the Italian adult population, as assessed by the Italian Nutrition Knowledge Questionnaire, has resulted in increased adherence to the MedDiet (45). Individuals showing high adherence to the MedDiet corresponded to those with the highest nutritional knowledge and, conversely, those with low adherence showed the lowest nutritional knowledge (45). However, greater knowledge or awareness of cardiovascular health and main CVD factors does not always translate into better self-care or risk perception, as demonstrated by a pilot observational study conducted on cardiovascular specialists during the 2022 National Conference of the Italian Society of Hypertension (53). Among 62 study participants, 19.4% were smokers, 17.7% had dyslipidemia, 26.3% had high blood pressure, and 11.3% had hypertension, of which 57.1% was uncontrolled (53). A non-adherence to guidelines-directed preventive measures for cardiovascular health was also evidenced (53). A multicenter cross-sectional observational study to assess the perception and knowledge of cardiovascular risk among Italian women also showed that good knowledge of the major cardiovascular risk factors was not associated with better recognition of CVD as a leading cause of death, and less than 10% of respondents perceived themselves as being at high CVD risk (54). However, increased CVD risk perception was associated with older age, a higher frequency of cardiovascular risk factors and disease, and a poorer self-rated health status (54).

## 5 Metabolic risk factors for CVD

Modifiable lifestyle/behavioral factors often contribute to common metabolic CVD risk factors, including high systolic blood pressure, elevated total cholesterol, elevated fasting glucose, and increased body mass index (5). The metabolic risk factors for CVD that are more prevalent among the Italian population are hypertension, dyslipidemias, T2DM and excess body weight (3).

### 5.1 Hypertension

Hypertension is a major contributor to the global burden of CVD and related mortality (55). In Italy, hypertension is quite frequent in the population. According to a nationwide opportunistic cross-sectional survey promoted by the Italian Society of Hypertension During the XVII World Hypertension Day in 2021, 42.3% of 1,354 volunteer participants aged 18–91 years reported being hypertensive (55). Among the hypertensive participants, 41.4% were taking medication and of these, 26.9% had controlled blood pressure (55). The prevalence of self-reported hypertension was higher in men (47.5%) than in women (38.4%), and increased with age (55). A similar survey organized in 2017 by the International Society of Hypertension in Italy consisting in 1 month of blood pressure screening in a larger number of participants found instead that 30.8% of the 10,076 volunteers aged ≥18 years were hypertensive (56). Of note is the decline in the prevalence of hypertension in the Italian population observed in the last two decades (57). Comparing data from HESs of adults aged 35–74 years conducted in Italy in 1998–2002, 2008–2012, and in 2018–2019 within the CUORE Project of the Istituto Superiore di Sanità, it was evidenced that systolic and diastolic blood pressure in men (136/86 mm Hg, 132/84 mm Hg; and 132/78 mm Hg) and in women (132/82 mm Hg, 126/78 mm Hg; and 122/73 mm Hg) significantly reduced (57). The same was observed for both the prevalence of raised blood pressure (50, 40, and 30% in men, and 39, 25, and 16% in women) and hypertension (54, 49, and 44% in men, and 45, 35, and 32% in women) with consistent trends according to age and educational level (57). In 2018–2019, hypertensive men and women with controlled blood pressure were 27 and 41%, but a significant favorable trend was also observed (57).

### 5.2 Abnormal lipid profile

Abnormal blood cholesterol is highly prevalent among the Italian population. According to data from the Longevity check-up 7+ (Lookup 7+) project, an initiative of health promotion campaigns conducted throughout Italy between 2016 and 2017, 64.5% of 3,040 participants (aged 18–98 years) had abnormal cholesterol levels, with no differences between women and men (34% vs. 36%) (58). Specifically, more than 40% of subjects had cholesterol levels between 200 and 240 mg/dL, and about 10% had cholesterol levels >240 mg/dL (58). Prevalence of abnormal cholesterol was higher among individuals aged 45–64 years (55% 200–240 mg/dL; 18% >240 mg/dL), and considering this age group the prevalence was higher in women than in men (77% vs. 62%) (58). Another study conducted between 2019 and 2020 involving subjects of a similar age range (45–59 y) from a population in central Italy, evidenced that both total cholesterol/HDL ratio (4.1 ± 1.1. vs. 3.5 ± 1.1) and triglycerides were higher in men than in women (129.1 ± 91.6 vs. 105.4 ± 62.8 mg/dL) (59). According to the 2008–2012 HES within the CUORE Project of the Istituto Superiore di Sanità, among 8,141 Italian subjects aged 35–74 years, 35.8% had hypercholesterolemia (34.7% of men and 36.8% of women) (1). The frequency of elevated LDL-C and hypertriglyceridemia reached approximately 68 and 23% among the population, with 67.8% of men and 67.6% of women having elevated LDL-C and 30.2% of men and 15.5% of women having hypertriglyceridemia (1).

### 5.3 Diabetes

The prevalence of T2D in Italy has increased over time consistent with population aging and in relation to the increase in obesity (60). Diabetes is one of the main cardiovascular risk factors and CVD is the leading cause of death among people with T2D in Italy (12). In the general Italian population, prevalence has increased from 3.8% in 2000 to 5.3% in 2016, or from 4.1 to 4.9% considering standardized prevalence controlled for the effect of population aging (60). Fasting blood glucose, hyperglycemia, and T2D prevalence have been shown to be related to educational level, which represents a proxy indicator of socioeconomic status (1, 60). For example, among women and men aged 65–74 years with a high school degree or higher, T2D prevalence was 6.8 and 13.2%. In contrast, among women and men of the same age and a lower educational level, it increased to 13.8 and 16.4%, respectively (60). On the other hand, T2D incidence in Italy is ~5 per 1,000 person-years. Incident cases of T2D accounted for ~10% of all cases detected in 2018. Incidence is higher in women than in men aged 11–40 years, but higher in men than in women >40 years (61).

### 5.4 Overweight and obesity

Considering data from the 2017–2018 TackSHS survey, the estimated overall prevalence of overweight and obesity in Italian adults aged ≥18 years was 44.0%, with overweight prevalence being 36.5% and obesity prevalence 7.5% (62). Similar prevalences were observed with data from the multiscope survey “Aspects of daily life” of the Italian National Institute of Statistics (ISTAT) conducted in 2015 on a representative sample of nearly 46,000 subjects, from which it emerged that 35.3% of the population had overweight and 9.8% had obesity (63). The 2020 version of the survey, although conducted with mixed survey technique (Computer Assisted Web Interviewing/Computer Assisted Personal Interviewing/Paper And Pencil Interviewing), confirmed that 36.1% of the adult population had overweight and 11.5% has obesity. Overall, 47.6% of the Italian population aged ≥18 years are overweight/obese (64). Excess weight was higher among men (64). In fact, 43.9% of men vs. 28.8% of women were overweight and 12.3% of men vs. 10.8% of women were obese. Both men and women aged 65–74 years showed the highest proportions of excess weight (64).

## 6 Conclusion and future direction

CVD remains the leading cause of mortality, morbidity, and disability in the Italian population, with IHD/CAD and stroke accounting for the most important CVD burden. Considering the multifactorial nature of CVD, major environmental, lifestyle/behavioral, metabolic and genetic risk factors involved in the development of the disease should be considered to establish effective prevention, treatment, and screening measures. Furthermore, given the pleiotropic effect of candidate genes, genetic variants with important frequency in each population should be considered to better assess CVD susceptibility, e.g., through population-targeted genetic risk scores (16, 46). Differences in CVD risk observed between populations may thus vary also according to genetic heterogeneity, making it important to consider population-specific interactions between frequent risk alleles, and environmental and cultural factors (8, 65, 66). Future studies should be focused on the identification of polymorphisms as prognostic and predictive biomarkers in CVD (67). Further research is also needed to shed light on the unclear mechanisms and processes underlying the interactions between the diverse CVD risk factors.

## Author contributions

CO-G: Conceptualization, Investigation, Writing – original draft, Writing – review & editing. EC: Investigation, Writing – original draft, Writing – review & editing. MB: Conceptualization, Validation, Visualization, Writing – review & editing. AA: Conceptualization, Resources, Supervision, Validation, Writing – review & editing.
