# Supplementary material for: Genetic, lifestyle and metabolic factors contributing to cardiovascular disease in the Italian population: a literature review
Source: Front Nutr. 2024 Apr 4;11:1379785. doi: 10.3389/fnut.2024.1379785 (PMC11024791; doi:10.3389/fnut.2024.1379785)
Supplement: Supplementary file 1 [file Table_1.DOCX]

Genetic, lifestyle and metabolic factors contributing to cardiovascular disease in the Italian population: a literature review

**Claudia Ojeda-Granados^1^, Elisabetta Campisi^1^, Martina Barchitta^1^, Antonella Agodi^1^**

^1^Department of Medical and Surgical Sciences and Advanced Technologies “GF Ingrassia”, University of Catania, 95123 Catania, Italy

| **Supplementary Table 1. Genotype and allele frequencies of genetic variants associated with CVD risk in the Italian population.** | | | | |
| --- | --- | --- | --- | --- |
| **Gene/locus** | **SNP names** | **rs number** | **Genotype and allele frequencies** | **Reference** |
| *CELSR2*  (1p13.3 locus) | G>T | rs629301 | TT = 0.69  GT = 0.28  GG =0.03  T = 0.83  G = 0.17 | [1] |
| *CDKN2A-CDKN2B*  (9p21.3 locus) | C>G | rs1333049 | CC = 0.32  CG = 0.50  GG = 0.18  C = 0.57  G = 0.43 | [2] |
| *APOC3* | -482C > T | rs2854117 | CC = 0.44  CT = 0.46  TT = 0.51  C = 0.67  T = 0.33 | [3] |
| *MMP3* | 1171 5A>6A | rs3025058 | 5A5A = 0.29  5A6A = 0.49  6A6A = 0.22  5A = 0.54  6A = 0.46 |  |
| *SELE* | G98T | rs1805193 | GG = 0.81  GT = 0.12  TT = 0.07  G = 0.87  T = 0.13 |  |
| 9p21.3 locus | C>G | rs1333049 | CC = 0.31  CG = 0.49  GG = 0.20  C = 0.56  G = 0.44 |  |
| *LRP8* |  | rs7546246, rs2297660, rs3737983,  R952Q  rs5177 | TACGC = 0.70 | [4] |
| *NPR3* | −55 C>A |  | Not possible to determine | [5] |
| *TCF7L2* | T>C | rs7901695 | TT = 0.40  CT = 0.44  TT = 0.10  T = 0.62  C = 0.38 | [6] |
|  | C>T | rs7903146 | CC = 0.43  CT = 0.40  TT = 0.17  C = 0.63  T = 0.37 |  |
|  | G>T | rs12255372 | GG = 0.40  GT = 0.45  TT = 0.15  G = 0.63  T = 0.37 |  |
| *TP53* | Arg72Pro | rs1042522 | Not possible to determine | [7] |
| *ENPP1* | K121Q | rs1044498 | Not possible to determine | [8] |
| 9p21.3 locus | C>T | rs1333040 | CC = 0.09  CT = 0.39  TT = 0.52  C = 0.29  T = 0.71 | [9] |
| *PECAM1* | V125L, | rs281865545 | LL = 0.25  VL = 0.49  VV = 0.26  L = 0.50  V = 0.50 | [10] |
|  | N563S |  | NN = 0.24  NS = 0.50  SS = 0.26  N = 0.49  S = 0.51 |  |
|  | G670R | rs1131012 | GG = 0.30  GR = 0.44  RR = 0.26  G = 0.52  R = 0.48 |  |
|  | 53 G>A |  | GG = 0.72  GA = 0.26  AA = 0.02  G = 0.85  A = 0.15 |  |
| *TNF* | -308 G/A | rs1800629 | GG = 0.74  AG = 0.24  AA = 0.02  G = 0.85  A = 0.15 | [11] |
| *CYBA* | C242T  (Tyr72His) | rs4673 | CC = 0.38  CT = 0.49  TT = 0.13  C = 0.63  T = 0.37 | [12] |
| *IL6* | -174 G>C | rs1800795 | GG = 0.33  GC = 0.42  CC = 0.25  G = 0.54  C = 0.46 | [13] |
| *ICAM1* | 469 E>K | rs5498 | EE = 0.19  EK = 0.52  KK = 0.29  E = 0.45  K = 0.55 |  |
| *MTHFR* | 677 C>T | rs1801133 | CC = 0.34  CT = 0.49  TT = 0.17  C = 0.58  T = 0.42 | [14] |
| *eNOS (NOS3)* | Glu298Asp | rs1799983 | Glu/Glu=0.42  Glu/Asp=0.45  Asp/Asp=0.13  Glu=0.65  Asp=0.35 | [15, 16] |
|  | T786 C | rs2070744 | TT=0.27  CT=0.52  TT=0.21  T=0.53  C=0.47 |  |
| *APOB* | XbaI | rs693 | XbaI = 0.39 | [17] |
| *APOE* | E2/E3/E4 | rs429358 and rs7412 | e2 = 0.05  e3 = 0.88  e4 = 0.07 |  |
| *LIPC* | T202T (C>G) |  | T202T |  |
| *ACE*  (17q23) | I/D 287-bp | rs1799752 | I/I = 0.24  I/D = 0.44  D/D = 0.32  I = 0.46  D = 0.54 | [18] |
| *AGTR1* | A1166C | rs5186 | AA = 0.47  AC = 0.44  CC = 0.09  A = 0.69  C = 0.31 |  |
| *ACE* | I/D 250-bp | rs1799752 | Not possible to determine | [19] |

**References**

1. Noto D, Cefalù AB, Martinelli N, et al (2021) rs629301 CELSR2 polymorphism confers a ten-year equivalent risk of critical stenosis assessed by coronary angiography. Nutr Metab Cardiovasc Dis 31:1542–1547. https://doi.org/10.1016/j.numecd.2021.01.018

2. Pignataro P, Pezone L, Di Gioia G, et al (2017) Association Study Between Coronary Artery Disease and rs1333049 Polymorphism at 9p21.3 Locus in Italian Population. J Cardiovasc Transl Res 10:455–458. https://doi.org/10.1007/s12265-017-9758-9

3. Vecoli C, Adlerstein D, Shehi E, et al (2014) Genetic score based on high-risk genetic polymorphisms and early onset of ischemic heart disease in an Italian cohort of ischemic patients. Thromb Res 133:804–810. https://doi.org/10.1016/j.thromres.2014.03.006

4. Shen G-Q, Girelli D, Li L, et al (2014) A novel molecular diagnostic marker for familial and early-onset coronary artery disease and myocardial infarction in the LRP8 gene. Circ Cardiovasc Genet 7:514–520. https://doi.org/10.1161/CIRCGENETICS.113.000321

5. Rubattu S, Giusti B, Lotta LA, et al (2013) Association of a single nucleotide polymorphism of the NPR3 gene promoter with early onset ischemic stroke in an Italian cohort. Eur J Intern Med 24:80–82. https://doi.org/10.1016/j.ejim.2012.09.002

6. Ciccacci C, Di Fusco D, Cacciotti L, et al (2013) TCF7L2 gene polymorphisms and type 2 diabetes: association with diabetic retinopathy and cardiovascular autonomic neuropathy. Acta Diabetol 50:789–799. https://doi.org/10.1007/s00592-012-0418-x

7. Gloria-Bottini F, Saccucci P, Magrini A, et al (2012) p53 codon 72 polymorphism and coronary artery disease: evidence of association with left ventricular ejection fraction. Am J Med Sci 343:127–130. https://doi.org/10.1097/MAJ.0b013e318223ac71

8. Bacci S, Rizza S, Prudente S, et al (2011) The ENPP1 Q121 variant predicts major cardiovascular events in high-risk individuals: evidence for interaction with obesity in diabetic patients. Diabetes 60:1000–1007. https://doi.org/10.2337/db10-1300

9. Ardissino D, Berzuini C, Merlini PA, et al (2011) Influence of 9p21.3 genetic variants on clinical and angiographic outcomes in early-onset myocardial infarction. J Am Coll Cardiol 58:426–434. https://doi.org/10.1016/j.jacc.2010.11.075

10. Listì F, Caruso C, Di Carlo D, et al (2010) Association between platelet endothelial cellular adhesion molecule-1 polymorphisms and atherosclerosis: results of a study on patients from northern Italy. Rejuvenation Res 13:237–241. https://doi.org/10.1089/rej.2009.0940

11. Sbarsi I, Falcone C, Boiocchi C, et al (2007) Inflammation and atherosclerosis: the role of TNF and TNF receptors polymorphisms in coronary artery disease. Int J Immunopathol Pharmacol 20:145–154. https://doi.org/10.1177/039463200702000117

12. Nasti S, Spallarossa P, Altieri P, et al (2006) C242T polymorphism in CYBA gene (p22phox) and risk of coronary artery disease in a population of Caucasian Italians. Dis Markers 22:167–173. https://doi.org/10.1155/2006/458587

13. Pola R, Flex A, Gaetani E, et al (2003) Synergistic effect of -174 G/C polymorphism of the interleukin-6 gene promoter and 469 E/K polymorphism of the intercellular adhesion molecule-1 gene in Italian patients with history of ischemic stroke. Stroke 34:881–885. https://doi.org/10.1161/01.STR.0000062346.70983.DF

14. Girelli D, Martinelli N, Pizzolo F, et al (2003) The interaction between MTHFR 677 C-->T genotype and folate status is a determinant of coronary atherosclerosis risk. J Nutr 133:1281–1285. https://doi.org/10.1093/jn/133.5.1281

15. Colombo MG, Andreassi MG, Paradossi U, et al (2002) Evidence for association of a common variant of the endothelial nitric oxide synthase gene (Glu298-->Asp polymorphism) to the presence, extent, and severity of coronary artery disease. Heart 87:525–528. https://doi.org/10.1136/heart.87.6.525

16. Colombo MG, Paradossi U, Andreassi MG, et al (2003) Endothelial nitric oxide synthase gene polymorphisms and risk of coronary artery disease. Clin Chem 49:389–395. https://doi.org/10.1373/49.3.389

17. Baroni MG, Berni A, Romeo S, et al (2003) Genetic study of common variants at the Apo E, Apo AI, Apo CIII, Apo B, lipoprotein lipase (LPL) and hepatic lipase (LIPC) genes and coronary artery disease (CAD): variation in LIPC gene associates with clinical outcomes in patients with established CAD. BMC Med Genet 4:8. https://doi.org/10.1186/1471-2350-4-8

18. Fatini C, Abbate R, Pepe G, et al (2000) Searching for a better assessment of the individual coronary risk profile. The role of angiotensin-converting enzyme, angiotensin II type 1 receptor and angiotensinogen gene polymorphisms. Eur Heart J 21:633–638. https://doi.org/10.1053/euhj.1999.1738

19. Arbustini E, Grasso M, Fasani R, et al (1995) Angiotensin converting enzyme gene deletion allele is independently and strongly associated with coronary atherosclerosis and myocardial infarction. Br Heart J 74:584–591. https://doi.org/10.1136/hrt.74.6.584
